# Structural basis for cooperativity of human monoclonal antibodies to meningococcal factor H-binding protein

**DOI:** 10.1038/s42003-019-0493-4

**Published:** 2019-06-26

**Authors:** Ilaria Peschiera, Maria Giuliani, Fabiola Giusti, Roberto Melero, Eugenio Paccagnini, Danilo Donnarumma, Werner Pansegrau, José M. Carazo, Carlos O. S. Sorzano, Maria Scarselli, Vega Masignani, Lassi J. Liljeroos, Ilaria Ferlenghi

**Affiliations:** 1 Centro National de Biotecnologia, 28049 Madrid, Spain; 2 GSK Vaccines Srl, 53100 Siena, Italy; 3 Department of Life Sciences, University of Siena, 53100 Siena, Italy; 4 Roche, 02180 Espoo, Finland

**Keywords:** Cryoelectron microscopy, Cryoelectron tomography, Meningitis

## Abstract

Monoclonal antibody (mAb) cooperativity is a phenomenon triggered when mAbs couples promote increased bactericidal killing compared to individual partners. Cooperativity has been deeply investigated among mAbs elicited by factor H-binding protein (fHbp), a *Neisseria meningitidis* surface-exposed lipoprotein and one of the key antigens included in both serogroup B meningococcus vaccine Bexsero and Trumenba. Here we report the structural and functional characterization of two cooperative mAbs pairs isolated from Bexsero vaccines. The 3D electron microscopy structures of the human mAb–fHbp–mAb cooperative complexes indicate that the angle formed between the antigen binding fragments (fAbs) assume regular angle and that fHbp is able to bind simultaneously and stably the cooperative mAbs pairs and human factor H (fH) in vitro. These findings shed light on molecular basis of the antibody-based mechanism of protection driven by simultaneous recognition of the different epitopes of the fHbp and underline that cooperativity is crucial in vaccine efficacy.

## Abstract (web summary)

Peschiera et al demonstrate the capability of four monoclonal antibodies from Bexsero vaccine to simultaneously bind the human factor H protein in vitro. These findings highlight the mechanism of cooperative antibody bindings and implications in vaccine efficiency.

## Introduction

N*eisseria meningitidis* is a worldwide etiological agent of severe diseases such as meningitis and septicemia. It is a gram negative diplococcus colonizing the nasopharynx of ~10% of healthy humans^1^. Although colonization is a common event, specific circumstances can lead to a local inflammation and a migration of the bacteria into the bloodstream resulting in acute disease, death, or permanent disability^1,2^. Crucial features in the steps of colonization, survival, and spreading are the bacterial strategies evolved to evade the immune system. One of these mechanisms lies in the capability to downregulate the complement pathway activation, through the binding of the meningococcal factor H-binding protein (fHbp) to the human factor H (fH), a soluble inhibitor of the alternative complement pathway^3^. FHbp is a surface-exposed lipoprotein of *N. meningitidis* expressed at different levels among the strains and genetically divided in three variants, var.1, var.2, and var.3^3,4^. FHbp binds fH on the bacterial surface, enabling the pathogen to evade alternative complement-mediated killing by the host innate immune system and to survive in human serum and blood^1,5^. The importance of fHbp in preventing meningococcal infection is reinforced by its presence as recombinant antigen in both vaccines against meningococcal serogroup B licensed so far, rLP2086 (in the US; Trumenba, Pfizer) and 4CmenB (in Europe, Canada, Australia, USA, and Brazil; Bexero, GSK). These vaccines were licensed based on their capability to elicit complement dependent, antibody-mediated bactericidal activity as measured by the serum bactericidal assay using human complement (hSBA)^1,3,4^. The ability of specific anti-fHbp human monoclonal antibodies (mAbs) to interact and augment protective immunity has been reviewed^6^, suggesting that nonbactericidal antibodies can cooperate and elicit serum bactericidal activity (SBA). Importantly, several publications focused on the characterization of murine anti-fHbp mAbs reveal that the majority is not able to be bactericidal alone, but elicited high bactericidal titers when coupled with another mAb anti-fHbp^6–16^. More recently, Giuliani et al.^17^ revealed that protective response mediated by the synergy of multiple bactericidal epitopes on the fHbp protein was demonstrated by antibody couples induced by 4CmenB in humans^17^. Indeed, the authors showed that couples of nonbactericidal mAbs simultaneously binding nonoverlapping regions of fHbp are functional in hSBA^17^. This result is in agreement with the idea that the ability of anti-fHbp mAbs to efficiently engage the C1q relies on the specific steric configuration assumed by the antibody-antigen complex^7,9,10^. In this perspective, the model of the C1 activation through hexameric IgG cluster proposed by Diebolder et al. supports with structural data this hypothesis^18^. Nevertheless, it has been shown that an increased susceptibility of bacteria to the complement-mediated bactericidal activity results from inhibition of the fHbp binding to fH^13^. Despite all the studies mentioned, the mechanism of cooperativity by which pairs of mAbs, individually nonbactericidal or showing low bactericidal activity, become bactericidal only when acting together still remains mostly unknown. The present work clarifies the structural and functional basis of the cooperativity of human mAbs coupled to an individual antigen using the recombinant mAbs generated from vaccine elicited antibodies^19^. Our results shed light on the mechanism of activation of the complement pathway by the simultaneous and stable binding of the cooperative human mAbs to different epitopes recognized on the antigen.

## Results

### Selection of cooperative and noncooperative mAbs

**Fig. 1:**
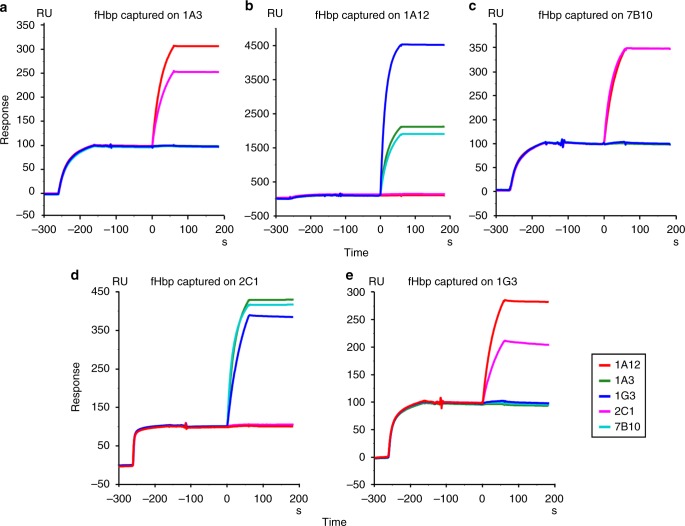
SPR binding competition analysis of cooperative and noncooperative fHbp–mAb complexes. The first SPR signal in each plot corresponds to the capturing of fHbp by the mAb coupled to the chip surface. The injection of the second mAb only produces an additional signal if cooperative couples are formed. Sensograms were normalized to the fHbp capture level (capture level = 100 RU in all the sensorgrams) to compensate for differences in capture efficiency on different mAbs. The color code of the second mAb is reported in the legend. **a** Immobilized mAb1A3. **b** Immobilized mAb1A12. **c** Immobilized mAb7B10. **d** Immobilized mAb2C1. **e** Immobilized mAb1G3. The profiles of immobilized mAb1A3 and 1G3 were reported also by Giuliani et al.^17^

The human IgG1 mAbs chosen for this study (1A3, 7B10, 1A12, and 2C1)^19^ possess specific characteristics as described in Giulani et al.^17^ Briefly, (1) high synergic bactericidal activity on H44/76 *N. meningitidis* (reference strain expressing fHbp var.1) in the presence of human serum as complement source^17,20^; (2) affinity to recombinant fHbp with dissociation constant (*K*_*D*_) values ranging from 1.72 E^−10^ M^−1^ to 0.9 E^−11^ M^−1^; and (3) different epitope localizations. mAb 1A12 was included in the panel as an example of a cross-reactive antibody binding to each of the three main variants of fHbp. A surface plasmon resonance (SPR) assay was used to discriminate between cooperative mAbs simultaneously binding fHbp and the noncooperative ones, binding mutually exclusive (Fig. 1). From data summarized in Supplementary Fig. 1, it is evident that increased SBA titers (as reported by Giuliani et al.) can be observed only when couples of mAbs can bind stably and contemporaneously to the same antigen. In the absence of simultaneous binding, no signal was present on the sensorgrams, indicating that the epitope recognized by the second mAb was not available for the binding when the protein was captured by the first mAb.

In the present study, each pair of selected mAbs is formed by an N-terminal-epitope-specific and a C-terminal-epitope-specific antibody. Although all the N-terminal-specific and C-terminal-specific mAbs were reported to recognize overlapping regions of their respective targeted domains, some differences were noticed and guided our selection. N-terminal-specific 7B10 and 1A3 were characterized by diverse affinities for the N-terminal domain, while a different degree of antigen specificity differentiates 2C1 and 1A12, with 1A12 able to recognize the C-terminal domains of all the three fHbp variants, while 2C1 resulted specific for fHbp variant 1 as described in Giuliani et al.^17^

### EM analysis of cooperative and noncooperative complexes

**Fig. 2:**
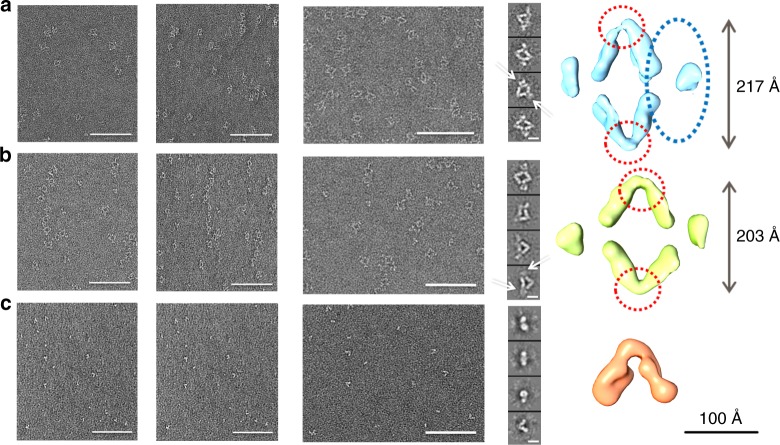
From NS-TEM images to 3D reconstruction of the immune complexes humAb–fHbp–humAb and of the huFab−fHbp−huFab. From left to right, the images in each panel describe the negative staining micrograph of RCT, untilted series, and representative 2D class averages and the surface views of the final 3D reconstruction. Blue dotted circles, in the final 3D reconstructions, indicate the mAb position in the complexes, while red dotted circles indicate the fHbp position. Rotation of the Fc portion of the mAbs is indicated by white arrows in the 2D class averages of the mAbs–fHbp complexes. **a** Complex formed by mAb7B10–fHbp–mAb2C1. **b** Complex formed by mAb1A3–fHbp–mAb1A12. **c** Complex formed by fAb7B10–fHbp–fAb2C1. The figures were generated by UCSF Chimera^21^. Scale bars, 100 nm for micrographs and 100 Å for both the class averages and the EM maps

Negative stain-transmission electron microscopy (NS-TEM) was used to visualize the morphology of the complexes formed by the cooperative mAbs with the fHbp (mAb7B10–fHbp–mAb2C1 and mAb1A3–fHbp–mAb1A12). These complexes regularly assumed the same rhomboidal assembly suggesting the formation of a stable tetrameric complex formed by two molecules of fHbp and two copies of mAb (Fig. 2a, b). The 2D class averages obtained, by applying the single-particle reconstruction method on the NS-TEM images, revealed that in both complexes, the mAbs were facing each other with the Fc portions pointing out the rhomboid and binding two molecules of fHbp, such that the two fHbp molecules are at the opposite corners of the rhomboid (Fig. 2a, b). Both the cooperative mAb-based complexes showed the Fc portions free to rotate (Supplementary Movie 1) and corresponded to the fuzzy electron density observed in the 2D classes (Fig. 2a, b). Moreover, to evaluate the role of the complementary-determining regions of the cooperative antibodies, the antigen-binding fragments (fAbs) of each mAb have been generated and TEM analysis performed on the fAbs–fHbp complexes. The corresponding 2D class averages assumed an overall “V” shaped structure (Fig. 2c) indicating that the complex was a heterotrimer made by the two fAbs each attached to its specific epitope of the same fHbp molecule (Fig. 2c). As expected the comparison of the 2D class averages of the fAb7B10–fHbp–fAb2C1 complex with the corresponding cooperative mAb7B10–fHbp–mAb2C1 complex revealed that the angle comprised between the different fAbs and the antigen was identical (~60°) (Supplementary Movie 2), different from the angle value (~74°) measured for the mAb1A3–fHbp–mAb1A12 complex. NS-TEM analysis applied to couples of mAbs recognizing the same domain (7B10–1G3 and 1A12–2C1) in presence of the fHbp molecule showed a relevant degree of heterogeneous structures with a high number of individual mAbs and some structurally irregular supramolecular complexes including rings (Supplementary Fig. 2).

### Structure comparison of two cooperative mAb complexes

As the two mAb–fHbp–mAb cooperative complexes and the fAb7B10–fHbp–fAb2C1 complex showed a preferred orientation on the TEM grids, it was convenient to apply the random conical tilt (RCT) approach^22^. Both untilted and tilted pair images were therefore collected and processed using the RCT protocol (Fig. 2a–c). The best 3D structures obtained as initial model for each immune complex were refined using 10,000 untilted particles generating a map of mAb7B10–fHbp–mAb2C1 with a resolution of 28 Å, a map of mAb1A12–fHbp–mAb1A3 with a resolution of 26 Å and a map of fAb7B10–fHbp–fAb2C1 at 23 Å resolution (FSC = 0.143) (Fig. 2a–c, right panel). In the cooperative complexes the distances between the two fHbp copies (positioned along the *Y*-axis) varied between 217 Å (mAb7B10–fHbp–mAb2C1) and 203 Å (mAb1A3–fHbp–mAb1A12). On the contrary, the Fc portions were free to rotate making impossible a detailed measurement (ranging in average from 250 to 260 Å) (Supplementary Fig. 3D). These values were in line with the model proposed by Biagini et al.^23^ where a distance of 300 Å between neighboring fHbp on the bacterial surface ensured an efficient C1q engagement. Both fHbp and mAbs densities are recognizable in the 3D reconstruction of each mAb–fHbp–mAb complexes (Fig. 2a, b, right panel) with the mAbs assuming the typical “Y” shape. The 3D reconstruction of the fAb7B10–fHbp–fAb2C1 complex (Fig. 2c, right panel) showed typical features, i.e., bilobed shape, of two fAbs attached to the density of the fHbp present at the vertex of the complex. A manual rigid-body fitting was performed using the crystallographic coordinates of IgG1 (PDB 1ZHZ) and fHbp (PDB 3VKD) separately (Supplementary Fig. 3). The coordinates of the fHbp were docked at the two vertices of the rhomboid architecture, while the coordinates of each fAb and Fc portions were fitted separately into the mAbs maps. FAbs were fitted in one of the elongated arms extending at the two sides of each of the two fHbp molecules. In every individual cooperative complex, the fAbs assumed a specific rotation along their *Z*-axis (Supplementary Fig. 3A–C). The electron density of the hinge region, corresponding to a flexible polypeptide linker that associates the fAbs and Fc portions of the antibody, is not clearly visible due to the low resolution of the maps and high flexibility of these regions.

The analysis of the antigen–antibody geometrical relationship was also performed comparing different reference-free 2D class averages for each complex. Both mAb–fHbp–mAb complexes assumed a rhomboid shape in which the fAb portions are tightly attached to the fHbp epitopes and resulted in a fixed geometry corresponding to a well-defined intensity in the 2D classes (Supplementary Fig. 4A, B). Moreover, in the mAb1A3–fHbp–mAb1A12 tetramer (Supplementary Fig. 4B), one of the mAb has both fAbs capable to rotate by 90° along their longitudinal axis. These rotations are observed neither in the mAb7B10–fHbp–mAb2C1 (Supplementary Movie 1) nor in the fAb7B10–fHbp–fAb2C1 complexes (Supplementary Fig. 4A, C). Indeed, the fAbs of different mAbs used the fHbp molecule as a pivot point that they rotate around their *Z*-axis thus resulting in fAbs that are viewed from their side (Fig. 2a, central panel) or on their back (Supplementary Fig. 4)

### Epitope mapping of cooperative and noncooperative mAbs

Absence of cooperative SBA is typically associated to the incapability of mAb pairs to simultaneously bind fHbp. To investigate if the absence of concurrent binding is caused by overlapping epitopes or by steric hindrance between mAbs, a fine epitope mapping by hydrogen deuterium exchange-mass spectrometry (HDx-MS) was performed. A total of 68 peptides, covering the 100% of fHbp sequence were monitored for their deuterium incorporation in the presence and absence of the different mAbs. The difference was considered relevant when the delta in the averaged value of deuterium incorporation was superior to 1 Da.

**Fig. 3:**
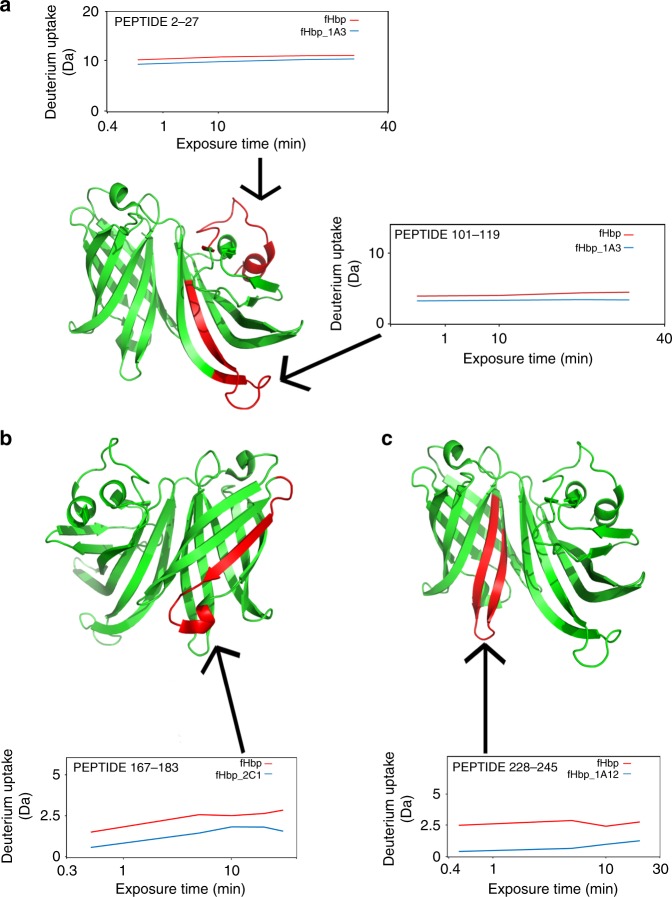
FHbp epitope recognition by HDX-MS. Each recognized epitope is labeled in red in the cartoon structure of the fHbp var.1 (PDB 3KVD). Boxes above and/or below the fHbp show the differential deuterium incorporation between the fHbp peptides in presence (blue) or absence (red) of mAb. Deuterium uptake was detected over time course (exposure time) ranging from 30 s to 30 min, and the peptide involved in the exchange is indicated by black arrows. Epitope mapping of cross-reactive mAb 1G3 is shown in Giuliani et al.^17^ **a** The conformational epitope recognized by the mAb1A3 is formed by a loop and a β-sheet segment both located onto the N-terminus part of the fHbp. The same conformational epitope was recognized by the mAb 7B10. **b** Epitope of the mAb2C1 is a segment located onto the C-terminus part of the fHbp and it is formed by a β-sheet. **c**, Epitope mapping of cross-reactive mAb 1A12 is shown in Lopez-Sagaseta et al.^20^

MAb1A3 and mAb7B10 presented overlapping epitopes composed by the segment of amino acids (aa) 2–27, match into the initial loop of the fHbp var.1, and the segment aa 101–119, corresponding to a β-strand (Fig. 3a). The epitope recognized by the mAb1G3 is precisely described in Giuliani et al.^24^ Briefly, the epitope is composed by the segment aa 2–27, which is in common with the other two mAbs, and the segment aa 43–70, equivalent to a long loop. We speculate that segment 101–119 formed the epitope recognized by 1A3 and 7B10 while the difference in deuterium incorporation observed at the N-terminal region 2–27, could be due to a stabilizing effect following the mAb interaction. The C-terminal β-barrel domain contained the epitope of the mAb2C1, which recognized the segment 167–183 corresponding mainly to a β-strand (Fig. 3b), and of the mAb1A12, as described by Lopez-Sagaseta et al.^20^

Collectively, our results provided a fine mapping of both N-terminal epitopes, recognized by 1A3, 1G3, and 7B10 human mAbs, and C-terminal epitopes, recognized by 2C1 and 1A12 human mAbs, respectively, and suggested that simultaneous binding by such mAbs is prevented by the overlapping localization of their epitopes. Moreover, our analysis evidenced that distinct loops are targeted at the C-terminal by 1A12 and 2C1 (Fig. 3). In this case the spatial proximity of these two epitopes appeared to be responsible of a steric hindrance that prevented their simultaneous binding.

### fH binding to the cooperative couples of human mAbs

**Fig. 4:**
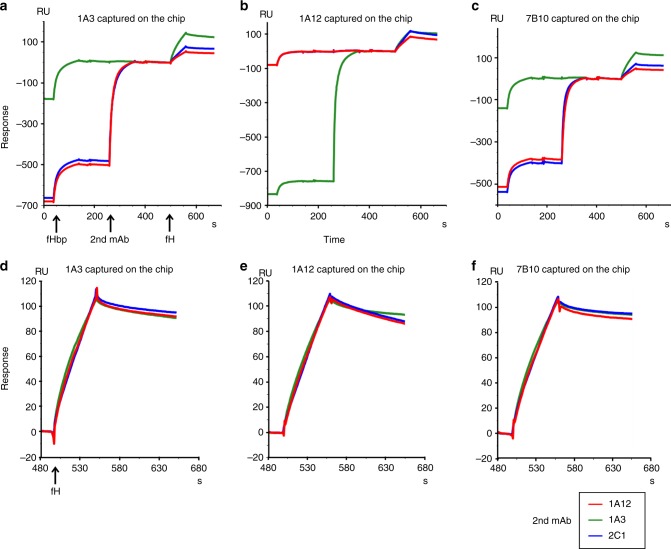
fH binding to cooperative complexes. The sensorgrams report the binding of fH to cooperative mAb–fHbp complexes. The first signal in each sensorgram corresponds to the capture of fHbp by the mAb coupled to the sensor chip; the presence of a second signal indicates the binding of the second cooperative mAb to the complex formed by the immobilized mAb and fHbp. Clearly, the second signal is absent when the second mAb is identical to the immobilized mAb; in these cases the corresponding epitope is already occupied by the immobilized mAb; the third signal demonstrates the binding of fH to the cooperative complex. Arrows below the time axis of **a** indicate injection start points for the various components participating in complex formation. The identity of the second mAb is given by the color code shown in the legend below the figure. **a**–**c** show the sensorgram profiles aligned at the fH baseline, while **d**–**f** report only the aligned and normalized fH signals. The mAb immobilized on the chip surface is indicated at the top of each panel

The differences in the repertoire of serum bactericidal antibodies against fHbp found between human or primate and mouse were previously attributed to the complex formed between fH and fHbp^19^. This binding, specific for human and primate fH, was hypothesized to restrict the available epitopes to fHbp regions outside the fH binding site^19^. To investigate the possible interference of the cooperative couples on the fHbp–fH binding, a biophysical analysis using SPR techniques was performed. After capturing fHbp on the sensor chip surface, the fHbp complexes with 1A3, 1A12, and 7B10 were tested for binding to human fH. The presence of an additional signal in the three profiles indicated that 1A3, 1A12, and 7B10 could form a ternary complex with the fHbp and the human fH (Supplementary Fig. 5A). Complex stabilities were assessed by determining their *k*_off_ rate constants (Table 1). While there is little difference of fH complex stability in the presence of mAbs 1A3 and 7B10, a notably faster rate of dissociation was detected in the presence of mAb1A12 (Table 1, Supplementary Fig. 5B). The next step consisted of performing the fH binding experiment in the presence of the cooperative complex already formed at saturating binding level conditions (Fig. 4). In this experiment, one mAb was coupled to the sensor to capture fHbp, while the second mAb was applied before the fH injection. Interestingly, the sensorgrams demonstrated the formation of quaternary complexes with all possible combinations of cooperative N-terminal and C-terminal targeting mAbs: a third signal was generated on the baseline of the stable cooperative complex profile (Fig. 4a–c). Again, complex stabilities were assessed by determining the *k*_off_ rate constants. While complexes involving the immobilized mAbs 1A3 and 7B10 show little difference in *k*_off_ rate constants when a second mAb is involved (Table 1), complexes formed with immobilized mAb 1A12 show higher variability in presence of a second mAb. In particular, the quaternary complex involving immobilized mAb 1A12 and captured 2C1 show considerable lower stability than the ternary complex with 1A12, while the quaternary complex comprising 1A12 and 1A3 is notably more stable than the ternary complex with 1A12 alone (Table 1, Fig. 4b).

**Table 1:** Dissociation rate constants of ternary and quaternary mAb–fHbp–fH complexes

**Immobilized mAb**	**1A3**			**1A12**			**7B10**		
**Ternary complex** ***k***_off_ (s^−1^)	7.4E−04			1.6E−03			3.2E−04		
**Second mAb**	1A12	(1A3)	2C1	(1A12)	1A3	2C1	1A12	1A3	2C1
**Quaternary complex** ***k***_off_ (s^−1^)	3.5E−04	7.4E−04	6.3E−04	1.8E−03	6.8E−04	4.3E−02	1.1E−04	3.2E−04	2.9E−04

Parentheses indicate where immobilized and second mAb are identical. In these cases, no binding signal for the second mAb was detectable and accordingly no important difference of *k*_off_ with respect to the ternary complex was observed

## Discussion

Cooperative bactericidal activity of mAbs is a biological mechanism that occurs when mAbs, which are individually not or weakly bactericidal, become bactericidal in combination. The underlying mechanism is still poorly understood, but plays a crucial role in mounting antibody-based protection induced by vaccination. In principle the ability by two different antibodies to bind simultaneously the same antigen increases the concentration of antibody Fc portions suitable for C1q recruitment and triggering of the classical complement pathway cascade, which ultimately results in bacterial killing. Beernink et al.^6^ observed bactericidal synergy in couples of murine mAbs anti-fHbp where at least one of the two mAbs was able to inhibit the binding between the fH and the antigen fHbp^6^ underlying the importance of downregulating the alternative pathway in the immune response against *N. meningitidis*. However, Vu et al. demonstrated that cooperative bactericidal activity may occur also combining mAbs that did not interfere with binding to fH^10^. The supposed key role of inhibition of fH binding has been further challenged by an important study on the human repertoire of antibodies elicited by fHbp from three human vaccinees, showing that bactericidal activity was provided also by human vaccine elicited anti-fHbp mAbs that do not inhibit the fH binding^19^. All these lines of evidence suggested that even in the presence of alternative complement pathway inhibition, the recruitment of C1q by anti-fHbp antibodies and the consequent activation of the classical complement pathway^9,12,25^ can still lead to efficient bacterial killing^7,9,12,26^. Overall our results suggest that antigens able to capture more than one antibody on their surface represent ideal candidates for a vaccine.

An optimal relative orientation of the two mAbs bound to the same antigen has been hypothesized as necessary a prerequisite for the recruitment of C1q^9,12,25^. In this work we focused exclusively on IgG1, the subclass known to be the most effective in the C1q activation^27^, that were produced as recombinant proteins using the heavy and light chains of variable regions from 4CMenB vaccine-elicited mAbs.

Our EM analysis, along with SPR data, clearly showed that cooperative human mAbs could form very stable quaternary complexes with fHbp. The complex assumed an overall rhomboid-shaped architecture with two fHbp molecules at two opposite vertices of the rhomboid and the two cooperative mAbs at the other two opposite vertices. Interestingly, the two cooperative couples of mAbs tested, although assembling into the same rhomboidal structure, presented different apertures of the angle formed by the antigen and the two fAbs attached, strictly related to the epitope position on the specific antigen binding sites. The geometrical relationship between the fHbp and the mAbs in the complex was not influenced by the antibody flexibility, as deduced from the 2D reference classes and the 3DEM maps of mAb–fHbp–mAb and fAb–fHbp–fAb complexes. Indeed, the angle formed between fHbp and the antibodies was similar (Fig. 2 and Supplementary Movie 1). Overall these observations suggest that epitope location is the driving force for the reciprocal orientation of the mAbs and the crucial feature for the cooperative phenomenon. It is interesting although to note that in both cooperative fHbp–mAbs complexes, the Fc portions are located around the same distance of ~250 Å. This distance is in line with the one that could be deduced by Mortenset al. between the globular heads at the opposite sites of C1q^28^. In a previous study Malito et al.^29^ reported that Fc portions of a couple of cooperative murine mAb bound to fHbp were separated by a distance of 130 Å, the same that separate two nonadjacent globular head of C1q in reported EM structure^24,28,30^. We can therefore speculate that a common feature among human and murine IgG cooperative couples bound to fHbp is the capability to accommodate the Fc in orientations highly adaptable to the C1q geometry.

The structures determined in the present study refer to free mAbs-based and fAbs-based complexes in solution, where antibodies can bind fHbp with both their antigen binding sites.

However, we cannot exclude a higher stoichiometric heterogenicity when the complexes are formed on the bacterial surface where the steric hindrance due to the presence of the membrane and of the other surface expressed proteins might play an important role. Nevertheless, the cooperative immune complexes are still able to recruit the C1q in vivo, activating the complement pathway as demonstrated by the high bactericidal titers reported in Giuliani et al.^17^

Interestingly, although Beernink postulated that inhibition of fH binding to fHbp was necessarily to observe cooperativity between fHpb-specific mAbs^25^, subsequent studies indicated that human mAbs isolated from vaccinees were able to cooperate despite their incapability to prevent fH binding^19^.

Accordingly, in this work we demonstrated that binding of pairs of cooperative antibodies do not prevent the fHbp interaction with fH, thanks to a possible spatial reorganization of the four molecules (mAb–fHbp–mAb–fH) present in the complex^29^.

Importantly, the high degree of heterogeneity observed by EM of the noncooperative human mAbs in the presence of fHbp and the results of the HDx-MS epitope mapping identified the partial epitopes overlapping or the steric hindrance of the mAb molecules as the main causes of the absence of complex formation. Taken together these data underline the fundamental role played by the epitope location in the mechanism of cooperative anti-fHbp mAbs activation.

Moreover, the identification of the amino acid residues affecting the location of the epitope could help in designing new vaccine candidates able to engage higher numbers of human mAbs^7,9^.

To our knowledge this is the first experimentally determined structure of cooperative vaccine elicited human mAbs in complex with the bacterial antigen fHbp which provides insights on the epitope protective properties. Our data underline the important role played by the synergistic mechanism between protective mAbs that recognize different epitopes present on the same protein antigen. The antibodies cooperativity together with the target antigen density on the bacterial surface^23^ are fundamental requirements for classical complement pathway activation and killing. For this reason, the understanding of the mechanism responsible of the synergic bactericidal activity could be used as a helpful information to assist the selection of vaccine candidates by prioritizing molecules able to stably engage more than one antibody simultaneously.

## Methods

### Cloning, expression, and purification of the fHbp

The recombinant protein fHbp var.1 full length (UniProt Q6QCC2) was expressed using the pET-21b plasmid (Novagen) in the *E. coli* strain BL21 (DE3) Star (Invitrogen) as previously published^4,31^. The purification was already reported^9^. Briefly, the fHbp growth was performed inducing the cells with 0.25 mM IPTG for 5 h at 25 °C. The recombinant protein was purified from the biomass using a sonication protocol followed by affinity chromatography (HisTrap HP, GE Healthcare) and cationic exchange chromatography step (HiTrap SP HP, GE healthcare) in 50 mM Tris, pH 8.0 buffer.

### Production and purification of human fAbs and mAbs

FAbs and mAbs anti-fHbp var.1 were produced according to the protocol described in Giuliani et al.^17^ Briefly, fAbs anti-fHbp var.1 corresponding to the cooperative mAb7B10 and to mAb2C1 were produced using heavy chain and light chain from immunoglobulin variable regions isolated from three vaccines. The expression was performed in *E. coli* strain Rosetta 2 (Novagen) in Enpresso B medium (Biosilta) and induced with 1 mM IPTG for 24 h at 25 °C. Following biomass harvest, lysis was performed in CelLytic Express (Sigma-Aldrich) buffer with 10 mM imidazole. The fAbs purified were obtained centrifuging the lysate at 18000 × *g* and subsequently using firstly an affinity chromatography (His Gravity Trap column, GE Healthcare) and secondly an ion exchange chromatography (HiTrap SP HP, GE Healthcare). To produce mAbs anti-fHbp var.1, the same heavy chain and light chain were optimized for mammalian expression and synthetized by GeneArt (Life Technologies), and the purified DNA products were ligated into pRS5a Igγ1, Igκ, and Igλ expression vectors (NIBR) containing the CMV proter and ampicillin resistance. The cloning was performed in *E. coli* DH5α cells. The recombinant mAbs were transiently produced using Expi293 cells (Life Technologies) and harvested at 3 and 6 days after transfection using a centrifugation at 900 × *g* for 10 min. Purification of mAbs was performed with protein G beads (GE Healthcare), according to manufacturer’s protocol and exchanged into PBS buffer.

### Selection of human mAbs

Since mAbs able to induce complement mediated killing of bacteria correlate with resistance to meningococcal meningitis^32,33^, the crucial characteristic of the mAbs was low or no bactericidal titer elicited when tested alone^17^, but high titer ranging from 512 to 2048 when coupled. The SBA titers of each mAb and pairs of mAbs were screened to find cooperative and noncooperative couples on H44/76 *N. meningitidis* (reference strain expressing fHbp var.1) using human serum as a complement source as reported by Giuliani et al.^17^ As a second criterion, a high affinity of each single mAb to the antigen was required to form stable complexes that could be characterized in solution without the use of cross-linking. SPR was used for detecting the parameters of the binding affinity and only mAbs showing dissociation constant (*K*_*D*_) values ranging from 1.72 E^-10^ M^−1^ to 0.9 E^-11^ M^−1^ were chosen. The third feature was the epitope location of each mAb determined by a combined approach of peptide array technology, peptide scanning analysis, and sequence alignment, as described by Giuliani et al.^17^ The noncooperative couples chosen are composed by one mAb that recognizes only the var.1 and one that recognizes all the three variants of the protein^17^.

### Complexes formation and purification

The complexes were generated by incubating the fHbp var.1 and the desired antibody or fAbs in a 1:1 molar ratio, for 1 h at room temperature and purified using a Superdex 200 Increase 3.2/300 (GE Healthcare).

### SPR for assessing cooperativity

SPR experiments were performed on a BIAcore T200 instrument (GE Healthcare). HBS-P (10 mM HEPES pH 7.4, 150 mM NaCl, 0.05% v/v Surfactant P20) was used as running buffer. Approximately 1000 RU of the desired mAb were immobilized on a CM-5 sensor chip using amine coupling chemistry, followed by injection of the recombinant fHbp var.1 (100 nM at 30 µl/min for a contact time of 1 min). The second mAb was injected subsequently at the same concentration and flow rate. Sensorgrams were analyzed using BIAcore T200 Evaluation 1.0 Software.

### SPR for competition assay for fH

The cooperative couples tested were mAb7B10–mAb2C1 and mAb1A3–mAb1A12. The same approach and conditions used for the discrimination between cooperative and noncooperative couples were applied. Capturing of fHbp var.1 was performed on the CM-5 sensor chip covalently loaded with mAb1A3, mAb1A12, or mAb7B10, followed by injection of second mAb to form the cooperative complex. fH was then injected with a concentration of 100 nM with a flow rate of 30 µl/min. The sensorgrams were analyzed as described above.

### HDX-MS analysis for epitope mapping

The HDX-MS analysis was performed according to Giuliani et al.^17^ Briefly the antibody/antigen complexes were formed by mixing 54 pmol of the selected antibody to an equimolar amount of recombinant fHbp var.1, incubated for 30 min at room temperature and prepared and analyzed as previously explained^9^. A control experiment for each complex was performed with the antigen alone at the same condition previously described, using PBS instead of the antibody. The difference was considered considerable when the delta in the averaged value of deuterium incorporation was superior to 1 Da.

### Transmission electron microscopy

Purified complexes were diluted to 0.03 mg/ml in 20 mM Tris, 300 mM NaCl, pH 8 buffer, and 2.5 µl were loaded for 30 s onto a 300 mesh carbon/formvar coated copper grid (Agar Scientific). After Blotting the excess, the grid was negatively stained with 1% aqueous uranyl acetate for 45 s and analyzed using a Philips CM200-FEG transmission electron microscope operating at 200 kV equipped with a TVIPS TemCam-F224HD CCD camera and TVIPS EM-Menu4, EM tools, and EM-SPC software packages. The micrographs were acquired at a magnification of 50,000×, corresponding to a pixel size of 3.3 Å/pixel on the specimen. As the complexes assumed a preferred orientation onto the grid, dataset of around 2000 untilted images and a set of 25 RCT pairs of images were collected at −55° and 0° for each sample.

### Image analysis and structure generation

The 2D analysis was performed on a subgroup of 10,000 particles extracted from the untilted dataset and subjected to 2D classification using Scipion-XMIPP3 with a CL2D method^34^. The 2D analysis allowed comparing the flexibility of the angle formed by the fAbs and the fHbp molecule in the different cooperative complexes (Supplementary Fig. 4). The RCT dataset was used to generate an initial model within Scipion Software^35^. Briefly, particles were independently picked from tilted and untilted datasets and tilt pairs were assigned according to Vilas et al.^36^ A subset of ~1000 tilt pairs was obtained and 2D classification was performed on the untilted particles using CL2D. RCT maps were then generated for each class average using the pairs extracted. The maps obtained for each immune complex, showing the best structural characteristic, were refined within Scipion-XMIPP3 projection matching^37^ using the subgroup of 10,000 particles extracted from the untilted dataset. The resolution was calculated at FSC = 0.143 criterion^21^. Each map was then manually fitted with the crystallographic coordinates of the immunoglobulin (PDB 1HZH) and of the fHbp molecule (PDB 3KVD) in Chimera^38^.

### Use of human sample and human data

ClinicalTrials.gov: NCT02305446. The purpose/aim of this Phase 3b study conducted in Poland was to assess the safety of a meningococcal group B vaccine and to collect blood donations to be used in furthering the development of vaccines against *N. meningitidis*. Healthy adults from 18 to 50 years of age received two doses of legacy novartis meningococcal group B vaccine given 2 months apart. Informed consent form was signed by all subjects.

### Reporting summary

Further information on research design is available in the Nature Research Reporting Summary linked to this article.

## Supplementary information

Supplementary Information

Description of Additional Supplementary Files

Supplementary Data 1

Reporting Summary

Supplementary Movie 1

Supplementary Movie 2

## Data Availability

The EM maps of the cooperative mAb–fHbp–mAb and fAb–fHbp–fAb complexes reported in this paper have been deposited in the Electron Microscopy Data Bank (accession no. EMD-4713, EMD-4714, and EMD-4715). Source data for Supplementary Fig. 3D are available in Supplementary Data 1.
