# Supplementary material for: Structural basis for cooperativity of human monoclonal antibodies to meningococcal factor H-binding protein
Source: Commun Biol. 2019 Jun 26;2:241. doi: 10.1038/s42003-019-0493-4 (PMC6595007; doi:10.1038/s42003-019-0493-4)
Supplement: Supplementary file 1 — Supplementary Information [file 42003_2019_493_MOESM1_ESM.pdf]

Supplementary information

Structural basis for cooperativity of human monoclonal antibodies to meningococcal factor H binding protein

Ilaria Peschiera[3], Maria Giuliani[1], Fabiola Giusti[1], Roberto Melero[3], Eugenio Paccagnini[2], Danilo Donnarumma[1], Werner Pansegrau[1], José M. Carazo[3], Carlos O. S. Sorzano[3], Maria Scarselli[1], Vega Masignani[1], Lassi J. Liljeroos[4], Ilaria Ferlenghi[1*]

[1]GSK Vaccines Srl, Siena 53100, Italy; [2] Department of Life Sciences, University of Siena, Siena 53100, Italy; [3]Centro National de Biotecnologia, Madrid 28049, Spain; [4] Roche, 02180 Espoo, Finland

To whom correspondence should be addressed: Dr. Ilaria Ferlenghi, GSK Vaccine Srl, Siena, E-mail: ilaria.x.ferlenghi@gsk.com

---

SUPPLEMENTARY FIGURE LEGEND:

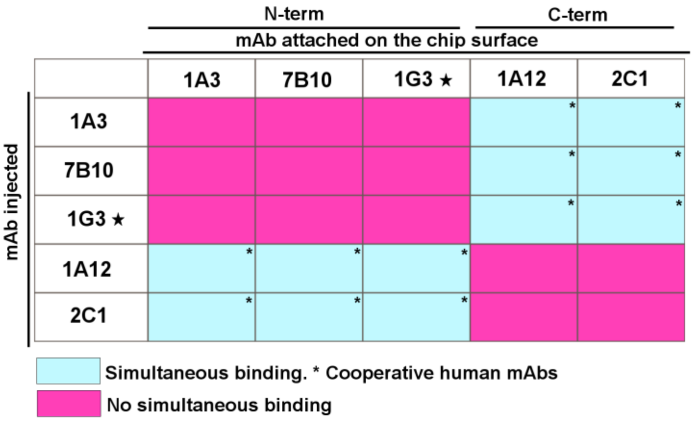

SUPPLEMENTARY FIGURE 1. Cooperative capability of human monoclonal antibodies tested by SPR technique. Simultaneous binding of human monoclonal antibodies on the same fHbp molecule analyzed by SPR technique. The mAbs attached on the chip surface are listed in the top row. The mAbs injected are listed in the left hand-side column. The human monoclonal antibodies able to bind simultaneously the fHbp antigen are colored in blue while those that are not able to bind simultaneously the fHbp are colored in red.1G3 SPR experiment have been shown in Giuliani *et al* 2018 (1).

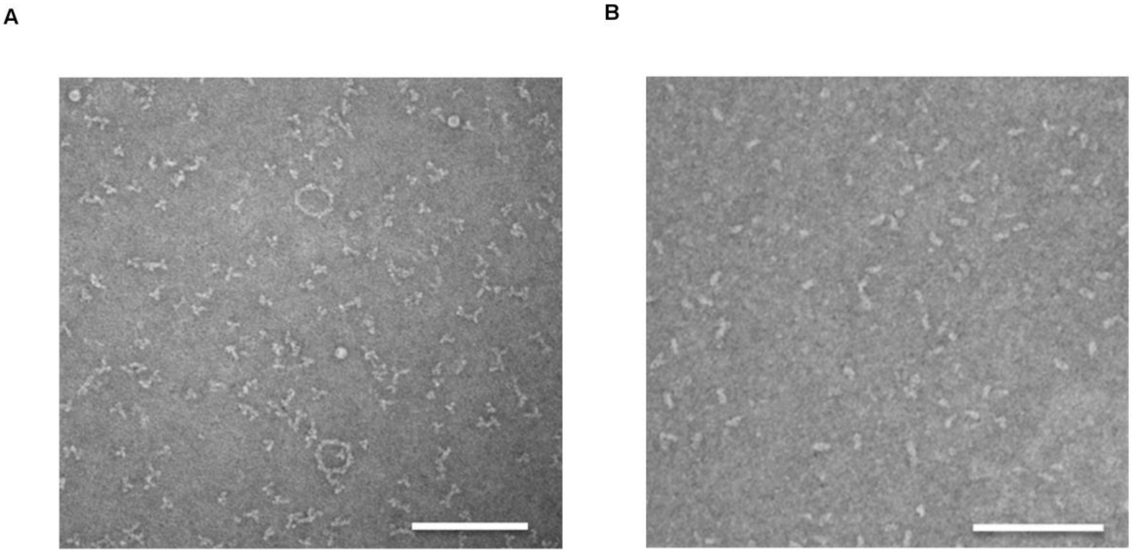

SUPPLEMENTARY FIGURE 2. Negative staining electron micrographs of the non-cooperative complexes of mAbs and mAbs distance distribution. A) mAb7B10-fHbp-mAb1G3 B) mAb1A12-fHbp-mAb2C1. Scale bars are 100 nm.

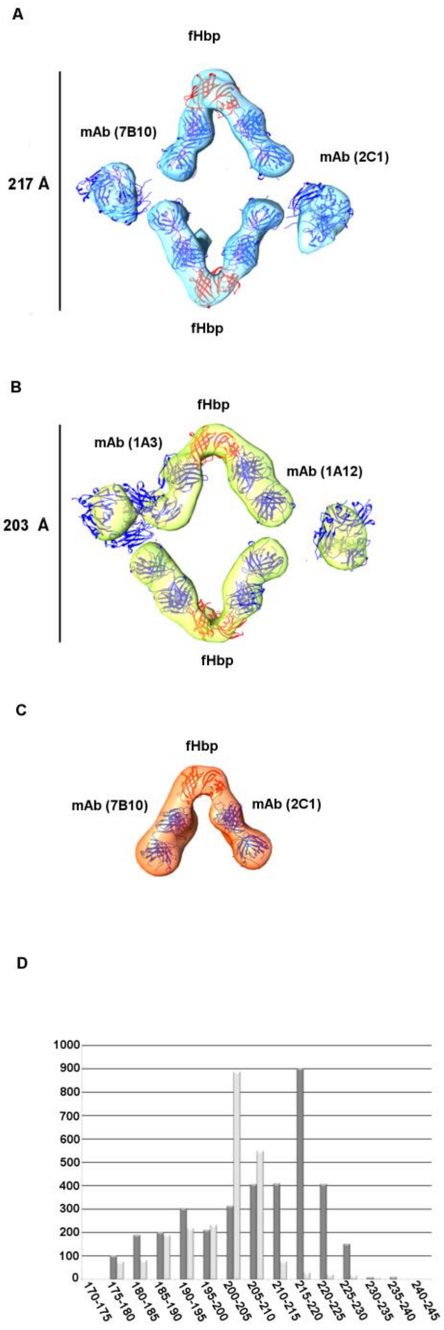

SUPPLEMENTARY FIGURE 3. RCT electron microscopy analysis of the mAb-fHbp-mAb and fAb-fHbp-fAb complexes. Side views of the 3DEM maps of the A) mAb7B10-fHbp-mAb2C1, B) mAb1A3-fHbp-mAb1A12 and C) fAb7B10-fHbp-fAb2C1complexes manually fitted with coordinates for the fHbp (red) (PDB 3VKD) and a general IgG1(light blue) (PDB 1HZH). D) FHbp regions distance distribution for mAb7B10-fHbp-mAb2C1 (light gray) (mean and +/-SD 217.28±11.67) and mAb1A3-fHbp-mAb1A12 (dark gray) (202.97±3.8)

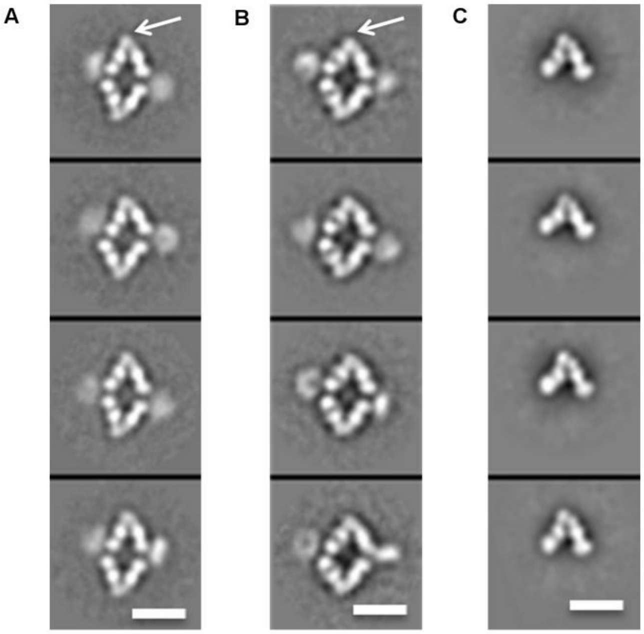

SUPPLEMENTARY FIGURE 4. Intramolecular fAb reciprocal orientation in the mAb-fHbp-mAb and fAb-fHbp-fAb complexes. Comparison of representative reference-free 2D class averages of two mAb-based complexes and one fAb-based complex. White arrows indicate the position of the fHbp molecule in all the complexes. While the value of the intramolecular fAb-fAb angle depends on the individual mAb, the value of the intermolecular fAb-fHbp-fAb angle is dictate by the position of the epitope. A) The complex of the cooperative tetramer mAb7B10-fHbp-mAb2C1-fHbp. B) The complex of the cooperative tetramer mAb1A3-fHbp-mAb1A12-fHbp and C) The cooperative trimer fAb7B10-fHbp-fAb2C1. Bars correspond to 100 Å.

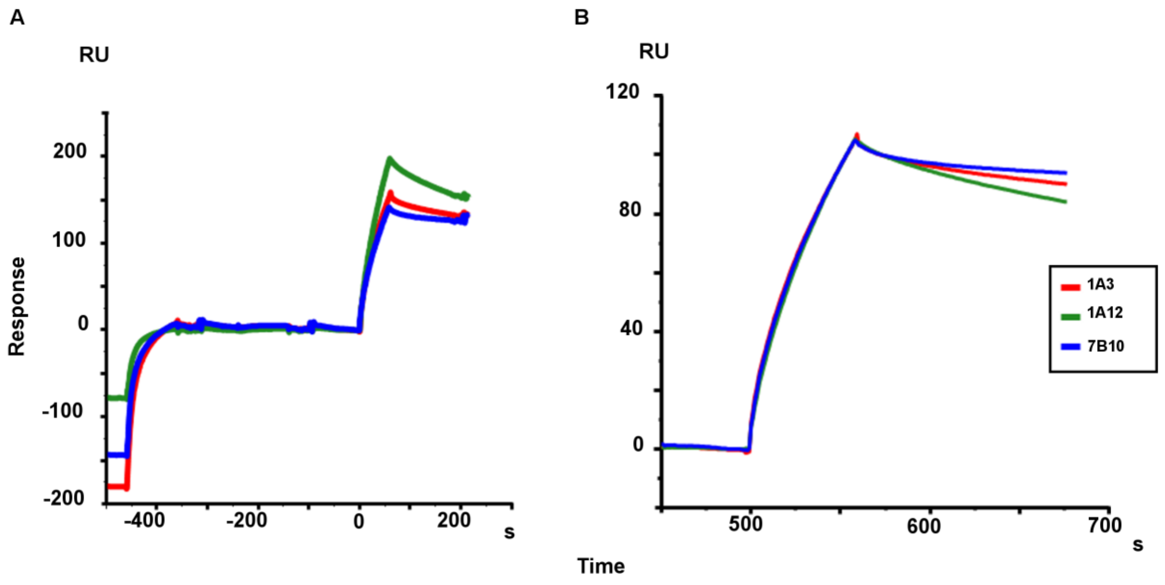

SUPPLEMENTARY FIGURE 5. FH binding analysis to the humAbs-fHbp complex. The sensorgram reports the profile of the fH binding on the binary complex formed by one humAb (mAb1A3, mAb1A12, mAb7B10) and fHbp, that was coupled to the sensor surface. A) The sensorgram profile is aligned at the sample baseline. B) Zoom of the binding profile of the fH to the mAb-fHbp complex normalized and aligned at the sample baseline. The color legend is reported in the panel B and indicates the mAb involved in the ternary complex (fHbp-mAb-fH).

REFERENCES

1. Giuliani, M., Bartolini, E., Galli, B., Santini, L., Lo Surdo, P., Buricchi, F., Bruttini, M., Benucci, B., Pacchiani, N., Alleri, L., Donnarumma, D., Pansegrau, W., Peschiera, I., Ferlenghi, I., Cozzi, R., Norais, N., Giuliani, M. M., Maione, D., Pizza, M., Rappuoli, R., Finco, O., and Masignani, V. Human protective response induced by meningococcus B vaccine is mediated by the synergy of multiple bactericidal epitopes. *Scientific reports* **8**, 3700 (2018)
