# Supplementary material for: Structural basis for cooperativity of human monoclonal antibodies to meningococcal factor H-binding protein
Source: Commun Biol. 2019 Jun 26;2:241. doi: 10.1038/s42003-019-0493-4 (PMC6595007; doi:10.1038/s42003-019-0493-4)
Supplement: Supplementary file 2 — Description of Additional Supplementary Files [file 42003_2019_493_MOESM2_ESM.docx]

**Description of Additional Supplementary Files**

**File Name**: Supplementary Data 1

**Description**: Excel file relative to graphic in Panel D of Figure 3 Supplementary materialFHbp regions distance distribution for mAb7B10-fHbp-mAb2C1 (light gray) (mean and +/-SD 217.28±11.67) and mAb1A3-fHbp-mAb1A12 (dark gray) (202.97±3.8)

**File Name**: Supplementary Movie 1

**Description**: Comparison of 2D class averages from two cooperative mAb-fHbp-mAb complexes. The video shows a comparison of the cooperative mAb7B10-fHbp-mAb2C1 with the cooperative mAb1A3-fHbp-mAb1A12 immuno-complexes.

The 2D class averages were generated by single particle method from NS TEM images.

**File Name**: Supplementary Movie 2

**Description**: Comparison of 2D class averages of the cooperative mAb7B10-fHbp-mAb2C1 with its corresponding fAb7B10-fHbp-fAb2C1complex. The 2D class averages were generated by single particle method applied on NS TEM images.
