# Supplementary material for: Structural basis for cooperativity of human monoclonal antibodies to meningococcal factor H-binding protein
Source: Commun Biol. 2019 Jun 26;2:241. doi: 10.1038/s42003-019-0493-4 (PMC6595007; doi:10.1038/s42003-019-0493-4)
Supplement: Supplementary file 4 — Reporting Summary [file 42003_2019_493_MOESM4_ESM.pdf]

nature research

Corresponding author(s): COMMSBIO-18-1085B

Last updated by author(s): May 17, 2019

Reporting Summary

Nature Research wishes to improve the reproducibility of the work that we publish. This form provides structure for consistency and transparency in reporting. For further information on Nature Research policies, see Authors & Referees and the Editorial Policy Checklist.

Statistics

For all statistical analyses, confirm that the following items are present in the figure legend, table legend, main text, or Methods section.

n/a	Confirmed	
☒	☐	The exact sample size (n) for each experimental group/condition, given as a discrete number and unit of measurement
☒	☐	A statement on whether measurements were taken from distinct samples or whether the same sample was measured repeatedly
☒	☐	The statistical test(s) used AND whether they are one- or two-sided<br>*Only common tests should be described solely by name; describe more complex techniques in the Methods section.*
☒	☐	A description of all covariates tested
☒	☐	A description of any assumptions or corrections, such as tests of normality and adjustment for multiple comparisons
☒	☐	A full description of the statistical parameters including central tendency (e.g. means) or other basic estimates (e.g. regression coefficient) AND variation (e.g. standard deviation) or associated estimates of uncertainty (e.g. confidence intervals)
☒	☐	For null hypothesis testing, the test statistic (e.g. F, t, r) with confidence intervals, effect sizes, degrees of freedom and P value noted<br>*Give P values as exact values whenever suitable.*
☒	☐	For Bayesian analysis, information on the choice of priors and Markov chain Monte Carlo settings
☒	☐	For hierarchical and complex designs, identification of the appropriate level for tests and full reporting of outcomes
☒	☐	Estimates of effect sizes (e.g. Cohen's d, Pearson's r), indicating how they were calculated

Our web collection on statistics for biologists contains articles on many of the points above.

Software and code

Policy information about availability of computer code

Data collection	TVIPS EM-Menu4, EM tools, EM-SPC software packages from TVIPS (www.tvips.com) are all softwares installed in the Philips CM200 FEG; EM-Menu4 software version 4.0.9.83 is installed in the TVIPS TemCam-F224HD CCD camera; EM Tools version 1.9.7.4 installed in the Philips CM200 FEG; EM-SPC (semi-automated data collection software for single Particle Work) installed in the Philips CM200FEG microscope (demo version installed by TVIPS).
Data analysis	web-based software for cryo-electron microscopy image processing Scipion-XMIPP3; http://scipion.cnb.csic.es/m/services/; software developed by Centro Nacional de Biotecnologia CNB-CSIC, Cantoblanco, Madrid,Spain.<br>Molecular graphics and analysis performed with USCF Chimera developed by the resource for biocomputing, visualization, and informatics at the University of California, San Francisco, with support from NIH P41-GM103311

For manuscripts utilizing custom algorithms or software that are central to the research but not yet described in published literature, software must be made available to editors/reviewers. We strongly encourage code deposition in a community repository (e.g. GitHub). See the Nature Research guidelines for submitting code & software for further information.

Data

Policy information about availability of data

All manuscripts must include a data availability statement. This statement should provide the following information, where applicable:

- Accession codes, unique identifiers, or web links for publicly available datasets
- A list of figures that have associated raw data
- A description of any restrictions on data availability

The EM maps of the cooperative mAb-fHbp-mAb and fAb-fHbp-fAb complexes reported in this paper have been deposited in the Electron Microscopy Data Bank (EMDB) (accession no. EMD-3646, EMD-3647 and EMD-3648). Each map was then manually fitted with the crystallographic coordinates of the immunoglobulin (PDB 1HZH) and of the fHbp molecule (PDB 3KVD)
Data present in Fig.3, in Video S1, Video S2, Fig.S3 and Fig.S4.

No restriction on data availability

Field-specific reporting

Please select the one below that is the best fit for your research. If you are not sure, read the appropriate sections before making your selection.

☒ Life sciences ☐ Behavioural & social sciences ☐ Ecological, evolutionary & environmental sciences

For a reference copy of the document with all sections, see nature.com/documents/nr-reporting-summary-flat.pdf

Life sciences study design

All studies must disclose on these points even when the disclosure is negative.

Sample size	no sample size performed
Data exclusions	no data exclusion performed
Replication	SPR experiments were performed at least three (3) times on the same sample
Randomization	no randomization was conducted due to the fact that there is no risk related to the experiments performed
Blinding	experiments were not conducted in blind due to the fact that there is no risk related to the experiments performed

Reporting for specific materials, systems and methods

We require information from authors about some types of materials, experimental systems and methods used in many studies. Here, indicate whether each material, system or method listed is relevant to your study. If you are not sure if a list item applies to your research, read the appropriate section before selecting a response.

Materials & experimental systems

n/a	Involved in the study	
☐	☒	Antibodies
☒	☐	Eukaryotic cell lines
☒	☐	Palaeontology
☒	☐	Animals and other organisms
☒	☐	Human research participants
☒	☐	Clinical data

Methods

n/a	Involved in the study	
☒	☐	ChIP-seq
☒	☐	Flow cytometry
☒	☐	MRI-based neuroimaging

Antibodies

Antibodies used	recombinant antibodies were produced and purified as described in Giuliani M. et al. (Scientific Report 2018, vol. 8(1):3700
Validation	Validation of primary antibodies performed as described in Giuliani M. et al (Scientific Report 2018, Vol 8(1):3700)
